# Safety of talimogene laherparepvec: a real‐world retrospective pharmacovigilance study based on FDA Adverse Event Reporting System (FAERS)

**DOI:** 10.1186/s40780-024-00388-0

**Published:** 2024-12-18

**Authors:** Yifan Hong, Kebin Cheng, Han Qu, Yuting Wang, Yuanyuan Wang, Guorong Fan, Zhenghua Wu

**Affiliations:** 1 School of Pharmacy, Shanghai Jiao Tong University, Shanghai, 200240 China; 2 Department of Clinical Pharmacy, School of Medicine, Shanghai General Hospital, Shanghai Jiaotong University, Shanghai, 200080 China; 3 Department of Respiratory and Critical Care Medicine, School of Medicine, Shanghai Pulmonary Hospital, Tongji University, 507 Zhengmin Road, Shanghai, 200433 China; 4 Department of Pharmacy, Shanghai Fourth People’s HospitalAffiliated to, Tongji University School of Medicine, Shanghai, China

**Keywords:** Oncolytic virus, Talimogene laherparepvec, Pharmacovigilance, FAERS database

## Abstract

**Background:**

Oncolytic virus therapy is a rapidly evolving emerging approach for the medical management of cancer. Talimogene laherparepvec (T-VEC) is the first and only Food and Drug Administration (FDA)-approved oncolytic virus therapy. Considering that exactly how T-VEC works is not known, there is a strong need for a comprehensive pharmacovigilance study to identify safety signals of potential risks with T-VEC.

**Objective:**

The objective of this study was to assess the risk of adverse events (AEs) related to T-VEC.

**Methods:**

We implemented a pharmacovigilance study utilizing individual case safety reports (ICSRs) reported to the FDA Adverse Event Reporting System (FAERS) database dated from 2004 quarter 1 to 2023 quarter 3. In this study, we used two algorithms, reporting odds ratio (ROR) and information component (IC), to assess the risk of AEs related to T-VEC.

**Results:**

A total of 1138 ICSRs of patients who received the T-VEC and reported to the FDA dated from 2004 quarter 1 to 2023 quarter 3 were available. A total of seven system organ classes (SOCs) demonstrated statistically significant signals, i.e. General disorders and administration site conditions, Injury, poisoning and procedural complication, Infections and infestations, Neoplasms benign, malignant and unspecified, Skin and subcutaneous tissue disorders, Hepatobiliary disorders, and Endocrine disorders. From the preferred term level perspective, the most reported AEs in T-VEC-treated patients were pyrexia, illness, influenza, influenza-like illness, and chills. Unexpected significant AEs were detected, such as sepsis, encephalitis, syncope, and lymphadenopathy.

**Conclusions:**

Most AEs in T-VEC-treated patients have been previously mentioned in the prescriptive information or documented in other clinical trials. But safety signals were also be detected in 4 unexpected AEs (sepsis, encephalitis, syncope, and lymphadenopathy). Further clinical trials need to be undertaken to facilitate a more comprehensive comprehension of the safety profile of T-VEC.

## Introduction

Oncolytic virus (OV) therapy is a rapidly evolving emerging approach for the medical management of cancer. It exploits the oncolytic ability of certain native viruses or genetically modified viruses to induce immune cell death by infecting and subsequently preferentially. lysing target cells (usually tumor cells) while leaving the patient’s normal human cells intact [1, 2]. A review by Kevin Harrington et al. showed that the capacity of OVs to induce antitumor immune responses (including but not limited to inducing immunogenic cell death) likely explains the primary mechanism responsible for the OVs mediated oncolytic activity [3]. Nonetheless, a significant proportion of OVs display inherent oncolytic activity against normal human cells, which can be further amplified by the introduction of viral mutations that render them capable of replicating exclusively within a malignant cellular environment [4, 5].

Talimogene laherparepvec (T-VEC) is a genetically modified oncolytic herpes simplex virus type 1 (HSV1) that has been developed for the treatment of unresectable recurrent melanoma [6–8]. It is the first and only Food and Drug Administration (FDA)-approved anti-cancer immunotherapy of its kind. The”Systemic Therapy for Melanoma: ASCO Guideline Update” recommended that for patients with unresectable melanoma who are not eligible or do not wish to pursue the recommended systemic therapies, T-VEC may be considered as a primary treatment option [9]. An open-label and multi-institutional randomized phase III study of 436 patients with unresectable melanomas conducted by Andtbacka et al. [6] showed that the durable response rate (DRR), the overall response rate (ORR), the median overall survival (OS) were significantly higher with T-VEC than the gene encoding human granulocyte macrophage colony-stimulating factor (GM-CSF). In addition to the previously mentioned, T-VEC also demonstrated improvements in time to treatment failure (TTF) and progression-free survival (PFS). In the subgroup analyses, the efficacy of T-VEC was most pronounced in patients with stage IIIB, IIIC, or IVM1a disease and in patients with treatment-naive disease. All these promising results about T-VEC ultimately led to full FDA approval in 2015.

Ever since it was initially approved by the FDA, T-VEC has been subjected to rigorous testing in a multitude of clinical trials and real-world studies, including in combination with immune checkpoint inhibitors (including but not limited to Ipilimumab and Pembrolizumab) [10] for patients with unresectable melanoma, in combination with immunotherapies in other types of solid tumors [11], and in real-world studies conducted by both single and multiple centers [12–15]. These pivotal clinical studies have confirmed both the safety and efficacy of T-VEC.

Considering that exactly how T-VEC works is not known, some serious adverse events (AEs) may remain undetected. During the routine clinical practice of T-VEC, many cases of some serious AEs associated with T-VEC therapy have been reported. For example, Brooks David et al. presented a case [16] of disseminated herpetic mucocutaneous infection and encephalitis after T-VEC injections. Therefore, there is a strong need for a comprehensive pharmacovigilance study to identify safety signals of potential risks with T-VEC. As one of the largest spontaneous and open-access adverse event databases, the USA FDA Adverse Event Reporting System (FAERS) database provides a broader perspective for drug surveillance. The retrospective pharmacovigilance study based on the FAERS database can offer informative insights into the safety profile of T-VEC in real-world clinical settings. In this study, we performed an observational, retrospective and disproportionality analysis to evaluate the AEs related to T-VEC based on the FAERS database for the purpose of early safety signal detection and stimulating further attention and investigation into related issues.

## Methods

### Study design and data sources

For this study, we implemented a retrospective pharmacovigilance study using the individual case safety reports (ICSRs) from the FAERS database dated from 2004 quarter 1 to 2023 quarter 3, the ICSRs are publicly available as quarterly data extract files on the FDA’s official website (https://fis.fda.gov/extensions/FPD-QDE-FAERS/FPD-QDE-FAERS.html). One of the limitations of the FAERS database is that cases may have multiple versions. This phenomenon is typically observed when the same AE is reported by multiple sources and assigned disparate case identification numbers. To remove these duplicates which may lead to spurious analytical results, we followed the approach proposed by Banda et al. [17] and extracted the most recent case version from all available follow-up cases. As this study utilized de-identified data, ethical approval was not required.

### Data extraction and identification

In the analysis of the safety of T-VEC, we first retrieved all ICSRs containing T-VEC as the primary suspected drug (PS). Then we searched the FAERS database for all T-VEC AEs reported between 2004 quarter 1 and 2023 quarter 3. The reports obtained were used for subsequent AE data mining and analyses. The reported AEs were coded by the preferred terms (PTs) from the standardized Medical Dictionary for Regulatory Activities 26.1(MedDRA), and we categorized PTs according to the system organ classes (SOCs).

### Statistical analysis

The use of quantitative signal detection in the analysis of spontaneous reports for drug safety assessments has been widely validated, demonstrating the efficacy of this approach in identifying potential risks associated with specific drugs [18, 19]. Adapting previous methods [20, 21] published in influential journals, a pharmacovigilance study was carried out to ascertain the risks of AEs related to T-VEC. In this study, we used two algorithms, reporting odds ratio (ROR) and information component (IC), to ascertain the correlation between T-VEC and AEs to avoid false positives. The ROR is a transparent familiar measure that is widely used in disproportionality analysis. Another advantage is that being an odds ratio, non-selective underreporting of a drug or ADR has no influence on the value of the ROR. Calculation of the IC using a Bayesian confidence propagation neural network was developed and validated by the Uppsala Monitoring Centre as a flexible, automated indicator value for disproportionate reporting that compares observed and expected drug–adverse event associations to find new drug–adverse event signals with identification of probability difference from the background data (full database) [22]. Probabilistic reasoning in intelligent systems (information theory) has proved to be effective for the management of large datasets, is robust in handling incomplete data, and can be used with complex variables. The information theory tool is ideal for finding drug–adverse event combinations with other variables that are highly associated compared with the generality of the stored data [23].

The statistical formula is as follows to calculate ROR and 95% confidence interval (CI),

$$ROR=\frac{ad}{bc}$$

$$95\%CI=\frac{ad}{bc}[{e}^{\pm 1.96\sqrt{\frac{1}{a}+\frac{1}{b}+\frac{1}{c}+\frac{1}{d}}}]$$

a: the number of target adverse events exposed to suspected drug.

b: the number of other adverse events exposed to suspected drug.

c: the number of target adverse events exposed to other drug regimens.

d: the number of other adverse events exposed to other drug regimens.

The statistical formula is as follows to calculate IC (95% CI),

$$\begin{array}{c}IC={\text{log}}_{2}\left(\frac{{\text{N}}_{\text{observed}}+ 0.5}{{\text{N}}_{\text{expected}}+ 0.5}\right)\end{array}$$

$$\begin{array}{c}{\text{N}}_{\text{expected}}=\frac{\left({\text{N}}_{\text{drug}}*{\text{N}}_{\text{effect}}\right)}{{\text{N}}_{\text{total}}}\end{array}$$

$$\begin{array}{c}{\text{IC}}_{025}={\text{log}}_{2}\left(\frac{{\text{N}}_{\text{observed}}+ 0.5}{{\text{N}}_{\text{expected}}+ 0.5}\right)-3.3*{\left({\text{N}}_{\text{observed}}+0.5\right)}^{-\frac{1}{2}}-2*{\left({\text{N}}_{\text{observed}}+0.5\right)}^{-\frac{3}{2}}\#\end{array}$$

$$\begin{array}{c}{\text{IC}}_{975}={\text{log}}_{2}\left(\frac{{\text{N}}_{\text{observed}}+ 0.5}{{\text{N}}_{\text{expected}}+ 0.5}\right)+2.4*{\left({\text{N}}_{\text{observed}}+0.5\right)}^{-\frac{1}{2}}-0.5*{\left({\text{N}}_{\text{observed}}+0.5\right)}^{-\frac{3}{2}}\#\end{array}$$

N_expected_: the number of case reports expected for the drug-adverse effect combination.

N_observed_: the actual number of case reports for the drug-adverse effect combination.

N_drug_: the number of case reports for the drug, regardless of adverse effects.

N_effect_: the number of case reports for the adverse effect, regardless of the drug.

N_total_: the total number of case reports in the database.

In essence, a positive signal can be interpreted as indicative of a higher incidence of AEs associated with the suspected drug when compared to one that would be expected by chance alone. The lower bound of ROR 95% CI (ROR_025_) is greater than 1.00 and the lower bound of IC 95% CI (IC_025_) is greater than 0, with the number of cases a is no less than 4 were used as a threshold for signal detection [18].

To exclude possibility that the signals are derived from concomitant ICIs, we conducted a disproportionality analysis to assess whether suspected AEs were differentially reported with a combination of T-VEC plus ICI compared with T-VEC in other drug regimens, including monotherapy.

The statistical formula is as follows to calculate ROR and 95% confidence interval (CI),

$$ROR=\frac{ad}{bc}$$

$$95\%CI=\frac{ad}{bc}[{e}^{\pm 1.96\sqrt{\frac{1}{a}+\frac{1}{b}+\frac{1}{c}+\frac{1}{d}}}]$$

a: the number of target adverse events exposed to T-VEC plus ICI combination.

b: the number of other adverse events exposed to T-VEC plus ICI combination.

c: the number of target adverse events exposed to the comparator (other ICI-containing regimens).

d: the number of other adverse events exposed to the comparator.

When the lower bound of ROR 95% CI is greater than 1.00 and the number of cases a is no less than 4, it implies that a significant disproportionality signal was detected and can be interpreted as statistically more adverse events observed for the drug/drug combination than one would except by chance alone.

Moreover, the patient demographics of AEs associated with T-VEC were summarized using descriptive analysis.

Data analysis was performed using R studio (Version 2023.12.1 + 402).

## Results

### Demographics description

During the time span encompassing from 2004 quarter 1 to 2023 quarter 3, a total of 1138 ICSRs of patients who received the T-VEC and reported to the USA FDA. The patient demographics of AEs related to T-VEC are summarized in Table 1. Most records were reported from the USA (78.6%). The number of reports involving men is close to the number of reports involving women. A total of 10.2% of patients in these cases died and 2.0% experienced a life-threatening situation. A total of 34.4% of reports pertained to elderly patients, defined as individuals with an age of at least 60 years.

**Table 1:** Patient demographics of AEs related to T-VEC in the FAERS database

Characteristic	Case N (%)
Total	1138
Sex	
Female	373(32.8%)
Male	401(35.2%)
Unknown	364(32.0%)
Reporting country	
USA	894(78.6%)
Rest of the world	243(21.3%)
Unknown	1(0.1%)
Reporting year	
2023 quarter 1 to 2023 quarter 3	71(6.2%)
2022	116(10.2%)
2021	115(10.1%)
2020	131(11.5%)
2019	151(13.3%)
2018	183(16.1%)
2017	194(17.0%)
2016	176(15.5%)
2015 and before	1(0.1%)
Age at onset	
< 18 years	3(0.3%)
18 ~ 44 years	54(4.5%)
45 ~ 59 years	167(14.7%)
≥ 60 years	391(34.4%)
Unknown	523(46.0%)
Outcome	
Death	116(10.2%)
Life-threatening	23(2.0%)
Hospitalization	234(20.6%)
Disability	11(1.0%)
Other serious events	437(38.4%)
Unknown	317(27.9%)

### Signal mining analysis at the system organ class level

Primarily, we conducted a signal mining analysis at the SOC level by comparing the T-VEC to all other drugs in the FAERS database. AEs associated with T-VEC at different SOCs are described in Table 2. A total of seven SOCs demonstrated statistically significant signals, i.e. General disorders and administration site conditions(*n* = 974; ROR 3.82 [two-sided 95% CI 3.24 ~ 4.51], IC 0.49 [two-sided 95% CI 0.39 ~ 0.57]), Injury, poisoning and procedural complications (*n* = 536; ROR 3.84 [3.42 ~ 4.32], IC 1.32 [1.18 ~ 1.43]), Infections and infestations (*n* = 410; ROR 3.36 [2.98 ~ 3.79], IC 1.32 [1.16 ~ 1.44]), Neoplasms benign, malignant, and unspecified (incl cysts and polyps)(*n* = 367; ROR 10.49 [9.26 ~ 11.88], IC 2.88 [2.70 ~ 3.00]), Skin and subcutaneous tissue disorders (*n* = 226; ROR 1.33 [1.15 ~ 1.54], IC 0.34 [0.12 ~ 0.50]), Hepatobiliary disorders (*n* = 27; ROR 2.68 [1.83 ~ 3.93], IC 1.36 [0.67 ~ 1.80]), and Endocrine disorders(*n* = 14; ROR 5.44 [3.21 ~ 9.21], IC 2.22 [0.99 ~ 2.76]). The record in General disorders and administration site conditions has the highest number and the record in Neoplasms benign, malignant, and unspecified (incl cysts and polyps) exhibits the most significant disproportionality according to the ROR.

**Table 2:** Signal mining analysis at the system organ class level

System organ class	N	ROR (95%CI)	IC (95%CI)
**General disorders and administration site conditions**	**974**	**3.82(3.24 ~ 4.51)**	**0.49(0.39 ~ 0.57)**
**Injury, poisoning and procedural complications**	**536**	**3.84(3.42 ~ 4.32)**	**1.32(1.18 ~ 1.43)**
**Infections and infestations**	**410**	**3.36(2.98 ~ 3.79)**	**1.32(1.16 ~ 1.44)**
**Neoplasms benign, malignant, and unspecified (incl cysts and polyps)**	**367**	**10.49(9.26 ~ 11.88)**	**2.88(2.70 ~ 3.00)**
**Skin and subcutaneous tissue disorders**	**226**	**1.33(1.15 ~ 1.54)**	**0.34(0.12 ~ 0.50)**
Gastrointestinal disorders	147	0.71(0.59 ~ 0.84)	-0.42(-0.70 ~ -0.23)
Nervous system disorders	110	0.49(0.40 ~ 0.60)	-0.89(-1.20 ~ -0.66)
Investigations	91	1.04(0.84 ~ 1.29)	0.06(-0.29 ~ 0.31)
Vascular disorders	90	0.94(0.76 ~ 1.16)	-0.08(-0.43 ~ 0.17)
Respiratory, thoracic, and mediastinal disorders	60	0.60(0.46 ~ 0.78)	-0.69(-1.11 ~ -0.38)
Musculoskeletal and connective tissue disorders	55	0.50(0.38 ~ 0.65)	-0.93(-1.38 ~ -0.61)
Renal and urinary disorders	41	1.10(0.81 ~ 1.51)	0.13(-0.39 ~ 0.50)
Metabolism and nutrition disorders	40	0.89(0.65 ~ 1.23)	-0.15(-0.68 ~ 0.22)
Blood and lymphatic system disorders	37	0.93(0.67 ~ 1.29)	-0.10(-0.64 ~ 0.29)
Psychiatric disorders	35	0.39(0.28 ~ 0.55)	-1.27(-1.83 ~ -0.87)
Cardiac disorders	32	0.56(0.39 ~ 0.80)	-0.79(-1.38 ~ -0.37)
**Hepatobiliary disorders**	**27**	**2.68(1.83 ~ 3.93)**	**1.36(0.67 ~ 1.80)**
Eye disorders	18	0.68(0.43 ~ 1.09)	-0.53(-1.31 ~ 0.02)
Surgical and medical procedures	18	1.29(0.81 ~ 2.06)	0.35(-0.45 ~ 0.90)
Immune system disorders	15	0.58(0.35 ~ 0.97)	-0.75(-1.60 ~ -0.14)
**Endocrine disorders**	**14**	**5.44(3.21 ~ 9.21)**	**2.22(0.99 ~ 2.76)**
Product issues	13	0.92(0.53 ~ 1.60)	-0.11(-1.04 ~ 0.53)
Social circumstances	8	1.79(0.89 ~ 3.59)	0.77(-0.54 ~ 1.55)
Ear and labyrinth disorders	7	1.13(0.54 ~ 2.39)	0.17(-1.15 ~ 1.02)
Reproductive system and breast disorders	2	4.93(1.23 ~ 19.76)	1.46(-2.94 ~ 2.40)

Bold text denotes significant positive signals

*ROR* Reporting odds ratio, *CI *Confidence interval

### Signal mining at the preferred term level

A total of 39 signals related to T-VEC were detected, and 22 signals were unearthed after excluding 17 signals not related to drugs such as cancer progression, tumor metastasis, and surgery. The 22 signals are presented in Table 3, in which the PTs were sorted by the corresponding SOCs and sorted in descending order by number of cases.

**Table 3:** Disproportionality analysis at the preferred term level

System organ class	Preferred term	N	ROR (95%CI)	IC (95%CI)
General disorders and administration site conditions	PYREXIA	118	6.61(5.46–80)	2.56(2.24–2.78)
	ILLNESS	78	23.41(18.6–29.47)	4.27(3.65–4.48)
	INFLUENZA LIKE ILLNESS	76	16.83(13.33–21.24)	3.85(3.30–4.08)
	CHILLS	62	9.87(7.64–12.75)	3.14(2.61–3.41)
	INJECTION SITE PAIN	39	2.57(1.87–3.54)	1.31(0.75–1.68)
	NECROSIS	19	60.28(38.27–94.94)	4.57(1.14–4.44)
	OEDEMA	18	6.12(3.84–9.76)	2.41(1.33–2.89)
	INJECTION SITE ERYTHEMA	18	2.66(1.67–4.24)	1.34(0.47–1.87)
	INFLAMMATION	14	2.31(1.36–3.91)	1.13(0.15–1.73)
	INJECTION SITE HAEMORRHAGE	13	3.10(1.79–5.36)	1.51(0.42–2.12)
	INJECTION SITE PRURITUS	10	2.76(1.48–5.14)	1.34(0.08–2.02)
Infections and infestations	INFLUENZA	78	7.70(6.12–9.69)	2.80(2.37–3.06)
	ORAL HERPES	42	42.82(31.45–58.29)	4.80(3.23–4.91)
	HERPES SIMPLEX	34	70.57(50.12–99.34)	5.11(2.54–5.02)
	HERPES VIRUS INFECTION	30	97.28(67.61–139.96)	5.22(1.92–4.98)
	CELLULITIS	22	6.86(4.50–10.46)	2.58(1.61–3.02)
	**SEPSIS**	**21**	**2.42(1.57–3.72)**	**1.21(0.43–1.71)**
	**ENCEPHALITIS**	**9**	**11.8(6.12–22.75)**	**2.90(0.43–3.33)**
Skin and subcutaneous tissue disorders	DERMATITIS	12	2.31(1.31–4.07)	1.12(0.05–1.77)
	SKIN LESION	10	6.36(3.41–11.85)	2.33(0.65–2.91)
Nervous system disorders	**SYNCOPE**	**27**	**3.89(2.65–5.69)**	**1.86(1.14–2.29)**
Blood and lymphatic system disorders	**LYMPHADENOPATHY**	**9**	**4.09(2.12–7.88)**	**1.81(0.29–2.47)**

Bold text denotes positive signals that are not mentioned in the prescribing information

*IC* information component, *ROR* reporting odds ratio, *CI* confidence interval

Overall, significant disproportionality signals were identified in 5 SOCs, of which general disorders and administration site conditions demonstrated the largest number of records. In the general disorders and administration site conditions, pyrexia (*n* = 118, ROR 6.61 [5.46–80], IC 2.56 [2.24–2.78]) was reported with the highest frequency. From the perspective of the preferred term level, the most reported AEs in T-VEC-treated patients were pyrexia, illness, influenza, influenza-like illness, and chills. Meanwhile, herpetic infection (including but not limited to oral herpes and herpes simplex) and injection site complications (including but not limited to necrosis and injection site erythema) were also significant disproportionality signals, i.e. oral herpes (*n* = 42, ROR 42.82 [31.45–58.29], IC 4.80 [3.23–4.91]), herpes virus infection (*n* = 30, ROR 97.28 [67.61–139.96], IC 5.22 [1.92–4.98]), injection site pain (*n* = 39, ROR 2.57 [1.87–3.54], IC 1.31 [0.75–1.68]), and cellulitis (*n* = 22, ROR 6.86 [4.50–10.46], IC 2.58 [1.61–3.02]). The record in herpes virus infection exhibits the most significant disproportionality according to ROR.

Except for AEs such as pyrexia, herpetic infection, and injection site complications, which were mentioned in the prescriptive information or documented in other clinical trials, four AEs not mentioned in the prescribing information were detected, including sepsis(*n *= 21, ROR 2.42 [1.57–3.72], IC 1.21 [0.43–1.71]), encephalitis(*n* = 9, ROR 11.8 [6.12–22.75], IC 2.90 [0.43–3.33]), syncope(*n* = 27, ROR 3.89 [2.65–5.69], IC 1.86 [1.14–2.29]), and lymphadenopathy(*n* = 9, ROR 4.09 [2.12–7.88], IC 1.81 [0.29–2.47]). They are presented in bold text in Table 3.

### Comparison of AEs risk between T-VEC in combination with immunotherapy and T-VEC in other drug regimens

Four ICIs (nivolumab and pembrolizumab, ipilimumab, atezolizumab) commonly used in combination with T-VEC were included in the analysis. The results are presented in Table 4. The majority of the detected signals diverge from the outcomes of the preceding disproportionality analysis. We found that the signal of sepsis (*n* = 4, ROR 11.09 [3.29—37.35]) was also detected in the cases with a combination of T-VEC plus nivolumab, which arises a bias that makes T-VEC have a higher ROR in sepsis.

**Table 4:** Comparison of AEs risk between T-VEC alone and in combination with ICIs

**Immune checkpoint inhibitors**	**Preferred term**	**N**	**ROR (95%CI)**
Pembrolizumab	MALIGNANT MELANOMA	9	2.83 (1.32-6.05)
	MALIGNANT NEOPLASM PROGRESSION	6	5.95 (2.15-16.42)
	RASH	6	3.24 (1.27-8.26)
	DYSPNOEA	5	5.40 (1.81-16.11)
	IMMUNE-MEDIATED ADVERSE REACTION	4	43.14 (4.78-389.59)
	AUTOIMMUNE COLITIS	4	21.55 (3.90-119.08)
	PLATELET COUNT DECREASED	4	8.60 (2.27-32.51)
	HYPOTENSION	4	4.28 (1.32-13.88)
Ipilimumab	DRUG INEFFECTIVE	6	12.03 (4.51-32.12)
	MALIGNANT NEOPLASM PROGRESSION	4	13.25 (4.06-43.3)
	MALIGNANT MELANOMA	4	4.21 (1.41-12.59)
	DEATH	4	3.02 (1.02-8.93)
Atezolizumab	CYTOKINE RELEASE SYNDROME	4	56.10 (13.14-239.51)
Nivolumab	OFF LABEL USE	13	2.16 (1.12-4.15)
	DRUG INEFFECTIVE	8	10.30 (4.31-24.61)
	DEHYDRATION	4	20.03 (5.21-77.08)
	SEPSIS	4	11.09 (3.29-37.35)

*ROR* reporting odds ratio, *CI* confidence interval

## Discussions

Additional therapeutic advances have changed the melanoma treatment landscape in recent years. Studies in real-world practice are therefore needed to understand the effectiveness and tolerability of T-VEC in a broader patient population. As far as we know, we report the first systematic pharmacovigilance database analysis that offered the most comprehensive account of AEs related to T-VEC based on the FAERS database. We systematically conducted a disproportionality analysis using ICSRs reported to the FAERS database, with a focus on the risk of AEs related to T-VEC.

According to the FAERS database, the majority of reporting regions are in the USA, which may be due to differences in population base comparing to other countries, such as Germany, and the fact that melanoma is the fifth most common cancer in the USA [24]. As a database of adverse event reports for drug and biologic products in the USA, FAERS is more likely to receive reports of AEs from the USA, which raises the possibility of reporting bias. Melanoma is particularly prevalent among white males, with an incidence (per 100,000) of 34.7 and 22.1 among white men and women [24], respectively. In this study, we didn’t see such gender disparity in the reported numbers, which may be due to the incomplete inputs. AEs are reported most frequently in people aged over 60 years, which is consistent with the epidemiology of melanoma. The mean age of diagnosis is 65, with 65.7% of diagnoses made in those ages 55 to 84 [24].

According to our retrospective study of 1,138 ICSRs of patients who received the T-VEC revealed that pyrexia, illness, influenza, influenza-like illness, and chills were the most reported AEs. Meanwhile, herpetic infection (including but not limited to oral herpes and herpes simplex) and injection site complications (including but not limited to necrosis and injection site erythema) were also significant disproportionality signals. Regarding herpetic infection and injection site complications, although the T-VEC drug label mentions them as important adverse reactions, our disproportionality analysis also showed significantly higher reporting of herpetic infection and injection site complications. Since those adverse events are potentially serious as previously reported [16], it is of vital importance to reassess the safety of the drug in relevant aspects. Overall, these AEs were generally by the instructions in the prescriptive information or consistent with previous reports. Nevertheless, this pharmacovigilance study revealed a disproportionately high reporting of sepsis, encephalitis, syncope, and lymphadenopathy associated with T-VEC. Although a fatal event associated with sepsis in the setting of *Salmonella* infection had been mentioned in the study by Andtbacka et al. [6], it was not described in detail or mentioned in the prescribing information. Hence, there is a compelling necessity for the implementation of well-conceived comparative safety clinical trials with the objective of validating the causal relationship, and it is imperative to contemplate the potential necessity for alterations to the prescriptive information to alert clinicians and patients to the possibility of the identified infections and lymphatic safety events. Although T-VEC has a novel mechanism of action, which arise a bias that makes it more likely to report AEs than other drugs. In view of the seriousness of its adverse effects, e.g., leading to termination of melanoma treatment, the results of our study continue to be of considerable importance.

The potential mechanism of AEs associated with T-VEC had not been described in an earlier study. Despite its selectivity, T-VEC is still a kind of HSV1 that infects humans. A theoretical issue with safety of OV therapy is the potential for the OVs to mutate and regain their pathogenic potential [25]. We hypothesized that it is for this reason that T-VEC causes a significantly higher risk of AEs to herpesvirus infection and injection site complications. In the case of disseminated herpetic mucocutaneous infection and encephalitis after T-VEC injections [16], following 1 therapy cycle, most of the cutaneous lesion, including skin away from injection site, presence of HSV-1 immunostaining from skin biopsy results, isolation of HSV-1 DNA from multiple symptomatic sites, and rapid improvement of mucocutaneous and encephalopathic symptoms with acyclovir aligned with true herpetic infection. The skin eruption progressed in case of the present patient while she was receiving treatment with broad-spectrum antibiotics and topical steroids that militated against alternative diagnoses of bacterial sepsis, cellulitis, and eczematous dermatitis reaction. Immune-related cutaneous adverse events were also unlikely, given that such reactions would not be expected to self-resolve during the short period witnessed in this case and would have been expected to demonstrate improvement with topical steroids. Back to the results of this pharmacovigilance study, Nervous system disorders and lymphatic system disorders may occur due to disseminated herpetic mucocutaneous infection and herpes simplex virus encephalitis. This explains why we detected a disproportionately high reporting of sepsis, encephalitis, syncope, and lymphadenopathy. The potential mechanism of sepsis, encephalitis, syncope, and lymphadenopathy associated with T-VEC had not been described in an earlier study. Further clinical trials need to be undertaken to support this hypothesis.

Some signals are also detected in the cases with immune checkpoint inhibitors (ICIs). It has possibility that these adverse events were triggered by ICIs and T-VEC might be just a concomitant drug. To exclude possibility that the signals are derived from concomitant ICIs, we conducted a disproportionality analysis to assess whether suspected AEs were differentially reported with a combination of T-VEC plus ICI compared with T-VEC in other drug regimens, including monotherapy. We calculated the ROR and 95% CI in patients who received the T-VEC plus ICI compared with T-VEC in other drug regimens (including monotherapy).

The majority of the detected signals diverge from the outcomes of the preceding disproportionality analysis. We found that the signal of sepsis was also detected in the cases with a combination of T-VEC plus nivolumab, which arises a bias that makes T-VEC have a higher ROR in sepsis. However, due to the small number of cases with a combination of T-VEC plus nivolumab, it may not change the results of the disproportionality analysis at the preferred term level. Overall, concomitant ICIs had a limited impact on the disproportionality analysis of T-VEC.

It must be acknowledged that this pharmacovigilance study is not without limitations, despite the advantages of data mining. Firstly, the ICSRs reported to the FDA are dependent on the quality of reporting, however, the FAERS database is heterogeneous in terms of the source of the reports. For example, deficiencies in the quality of the data, such as incomplete inputs, have been identified as a potential source of bias in the analysis [26]. Secondly, we were unable to modify our analysis of potential confounding variables, it was not possible to obtain further clinical information from the FAERS database, such as prior treatment regimens, which may influence the subsequent evaluation of disproportionate signals for AEs. By focusing on the “primary suspect” drug and conducting a disproportionality analysis about concomitant ICIs, we substantially refined our analytical scope, eliminating extraneous background noise and heightening the specificity of our results. The signals discerned may possibly reflect the true link between the drug and the observed adverse event [27]. Thirdly, it is important to note that the FDA receives reports of only a portion of all adverse events associated with pharmaceutical agents. Consequently, it is not possible to draw definitive conclusions from such disproportionality analyses, which rely on such databases. And It is imperative that our findings need to be validated in prospective clinical trials. Nevertheless, as one of the largest databases for open-access post-marketing drug event data globally, the FAERS database offers the potential to identify associations between suspected drugs and AEs in real-world clinical settings. This disproportionality analysis of the safety of T-VEC based on real-world data suggests the existence of critical issues of T-VEC that could offer informative insights for future clinical trials.

## Conclusions

In conclusion, we conducted a pharmacovigilance study using the FAERS database to investigate the relationship between T-VEC and AEs from various perspectives, with the potential risks identified and quantified. Significant signals of several clinically important AEs were detected in the disproportionality analysis. Most AEs in T-VEC-treated patients have been previously mentioned in the prescriptive information or documented in other clinical trials. But significant safety signals were also be detected in 4 unexpected AEs (sepsis, encephalitis, syncope, and lymphadenopathy). Further clinical trials need to be undertaken to corroborate the findings of this study. These endeavors will facilitate a more comprehensive comprehension of the safety profile of T-VEC, thereby aiding in the formulation of optimal clinical decisions regarding the utilization of T-VEC in the treatment of unresectable melanoma or other solid tumors.

## Data Availability

All data relevant to the study are included in the article or uploaded as online supplemental information.

## Abbreviations

T-VEC: Talimogene Laherparepve
AE: Adverse event
ICSR: Individual case safety report
FDA: Food and Drug Administration
SOC: System organ class
PT: Preferred term
ROR: Reporting odds ratio
IC: Information component
FAERS: FDA Adverse Event Reporting System
OV: Oncolytic virus
ASCO: American Society of Clinical Oncology
HSV1: Herpes simplex virus type 1
CI: Confidence interval
DRR: Durable response rate
ORR: Overall response rate
OS: Overall survival
GM-CSF: Gene encoding human granulocyte macrophage colony-stimulating factor
TTF: Time to treatment failure
PFS: Progression-free survival
PS: Primary suspected
MedDRA: Medical Dictionary for Regulatory Activities
ICIs: Immune checkpoint inhibitors
